# Effects of a Functional Food Made with *Salvia hispanica* L. (Chia Seed), *Amaranthus hypochondriacus* L. (Amaranth), and an Ethanolic Extract of *Curcuma longa* L. (Curcumin) in a Rat Model of Childhood Obesity

**DOI:** 10.3390/foods13111720

**Published:** 2024-05-30

**Authors:** Gloria Manuela Rivero-Salgado, Sergio Roberto Zamudio, Tomás Alejandro Fregoso-Aguilar, Lucía Quevedo-Corona

**Affiliations:** Departamento de Fisiología, Escuela Nacional de Ciencias Biológicas, Instituto Politécnico Nacional, Av. Wilfrido Massieu 399, Nueva Industrial Vallejo, Gustavo A. Madero, Mexico City 07738, Mexico; gloriaqfi@gmail.com (G.M.R.-S.); szamudio@ipn.mx (S.R.Z.); tfregoso@ipn.mx (T.A.F.-A.)

**Keywords:** childhood obesity, rat model, chia seed, amaranth, curcumin

## Abstract

Obesity is a global health problem and is increasing in prevalence in most countries. Although obesity affects all age groups, children are the most vulnerable sector. Functional foods are novel formulated foods containing substances (i.e., nutrients, phytochemicals, probiotics, etc.) that have potential health-enhancing or disease-preventing value. The research objective was to study the possible beneficial effects of providing a functional food made with amaranth flour, chia seed, and curcumin extract on the metabolism and behavior of a rat model of childhood obesity. Male Wistar rat pups from two litters of different sizes, a normal litter (NL) (10 pups) and a small litter (SL) (4 pups), were used. After weaning, the rats were fed a hypercaloric diet (HD) or an HD supplemented with the functional food mixture. Body weight and energy intake were measured for seven weeks, and locomotor activity, learning, and memory tests were also performed. At the end of the experiment, glucose and lipid metabolism parameters were determined. The results showed that in this model of obesity produced by early overfeeding and the consumption of a hypercaloric diet, anxiety-like behaviors and metabolic alterations occurred in the rat offspring; however, the provision of the functional food failed to reduce or prevent these alterations, and an exacerbation was even observed in some metabolic indicators. Interestingly, in the NL rats, the provision of the functional food produced some of the expected improvements in health, such as significant decreases in body weight gain and liver cholesterol and non-significant decreases in adipose tissue and leptin and insulin serum levels.

## 1. Introduction

Obesity is a worldwide health problem and is increasing in prevalence in most countries. The World Health Organization (WHO) reported that in 2016, an estimated 39% of adults presented with overweight, 13% presented with obesity, and around 8% of children and 29% of teenagers presented with either obesity or overweight [1]. The major causes of this alarming epidemic are the ingestion of high quantities of saturated fats and added sugars in the diet and a reduction in physical activity [2]. Although obesity affects men and women in all age groups, children represent the most vulnerable sector of the population. This is because, in infancy, children acquire their eating behavior [3], making them more prone to an obesogenic environment that promotes the ingestion of foods and beverages with high sugar content and energy density and a sedentary lifestyle. Moreover, it has been estimated that three-quarters of children who are overweight or obese will continue to suffer from this condition into adulthood, thus having an increased risk of developing non-communicable diseases (NCDs) later in life, such as cardiovascular diseases, type 2 diabetes mellitus, non-alcoholic fatty liver disease (NAFLD), dyslipidemia, musculoskeletal disorders, and some types of cancer [3,4]. These data indicate that it is particularly important to study the mechanisms of obesity and make efforts to prevent excessive body weight gain from occurring during childhood in order to reduce the prevalence of obesity and the long-term consequences of the high-risk cardiometabolic events that affect a large proportion of the people with these conditions [5].

One animal model designed to study the impact of childhood obesity was obtained by reducing the litter size from normal (eight to ten rat pups) to small (three to four offspring per dam) during the lactation period. This enables the offspring rat to suckle a higher quantity of milk when raised in a small litter (SL) compared with a normal litter (NL), resulting in a greater body weight gain [6,7,8] that is exacerbated after weaning when the animals receive high-density energy diets [9]. The overnutrition, overweight, and rapid growth that are produced in the infant rat under these conditions have a great influence on the health and metabolism of the adult animal [10,11], producing an increase in the prevalence of obesity, type 2 diabetes, metabolic syndrome, dyslipidemia, hypertension, and NAFLD developing in adulthood. Hence, the development of the hypothesis that the programming of a health state or the development of metabolic diseases later in life has an early origin [7,12].

Although the first proposal for preventing obesity is a healthy environment with a balanced diet and adequate physical activity, the changes in lifestyle that have occurred in recent decades toward more sedentary activities and the ingestion of low-quality, high-energy-dense processed foods suggest that an enhancement of foods with beneficial ingredients is needed.

Functional foods are foods with beneficial health effects in addition to their nutritional properties. This common definition is very broad and explains the concept of functional food poorly. Recently, Temple [13] proposed a new rational definition: functional foods are novel formulated foods that contain substances (i.e., nutrients, dietary fiber, phytochemicals, probiotics, etc.) or live microorganisms that have potential health-enhancing or disease-preventing value. These foods can be processed, and bioactive substances with known physiological effects can be added to reinforce their beneficial properties [14]. For instance, the satiating effect conferred by the ingestion of proteins is linked to its thermogenic effect (increase in metabolic rate) and the secretion of satiating gastrointestinal hormones such as glucagon-like peptide 1 (GLP-1) and peptide tyrosine-tyrosine (PYY) by the intestinal cells [15]. Dietary fiber also has a beneficial effect against dyslipidemia, which occurs in obesity [16].

To formulate a functional food with the aim of decreasing the occurrence of obesity and the associated biochemical, metabolic, and behavioral alterations present in a rat model of childhood obesity, we produced a mixture of amaranth grain flour (*Amaranthus hypochondriacus* L.), which comprised a good proportion of essential amino acids such as leucin in its proteins [17], and ground chia seeds (*Salvia hispanica* L.), owing to its high protein and polyunsaturated fat contents, with alpha-linolenic acid (ALA) as its main component [18]. Additionally, amaranth and chia contain high quantities of total dietary fiber, minerals, and vitamins. Furthermore, an ethanolic extract of *Curcuma longa* L., which contains curcumin, the major polyphenolic active component present in the extracts of the rhizome, was added to the mixture, which was incorporated into the hypercaloric diet provided to rats. These ingredients were chosen because there have been reports of their beneficial effects against obesity or the associated alterations in metabolism.

Amaranth, owing to its high leucin content, has a potential satiating effect, thus preventing diet-induced obesity and modulating insulin signaling in muscle cells and glucose homeostasis [15,19,20,21]. The intake of chia seed reduces adiposity in subjects who are overweight orobese [22] and rats [23,24], effects that have been associated with its high proportion of ALA (55–64%), an omega-3 polyunsaturated fatty acid [18]. In rats, chia restores insulin sensitivity, improves glucose tolerance, lowers adiposity, and restores serum triglycerides (TG), free fatty acids (FFA), and cholesterol to normal values [24]. Moreover, curcumin significantly reduced obesity and metabolic disorders in a model that mimicked childhood obesity in rats [25]. Turmeric and its ethanolic, hexanic, methanolic, and aqueous extracts have been found to be effective against obesity and metabolic syndrome [26,27,28].

## 2. Materials and Methods

### 2.1. Animals

A total of 12 female rats weighing 200–250 g and 6 male rats weighing 250–300 g, both of the Wistar strain, were used. The animals were housed in collective cages in a chamber at a temperature of 22 ± 1 °C, with 55 ± 5% relative humidity, light/dark cycles of 12 h, and access to tap water and food ad libitum. All experiments on animals were carried out following the ethical recommendations for the proper use and housing of animals, based on the Norma Oficial Mexicana [29] and the National Institutes of Health Guide for the Care and Use of Laboratory Animals [30].

### 2.2. Experimental Design

After 10 days of habituation to the allocation in our facilities, female rats were mated with male rats, and after 4 days, the female rats were individually housed in breeding cages and fed standard chow ad libitum during gestation and lactation.

On the day of parturition, each litter was randomly assigned as a normal litter (NL) or a small litter (SL) to form the different experimental groups. NLs were adjusted to 10 offspring rats (4–6 females and 4–6 males) on postnatal day (P) 2. SLs were adjusted to 6 offspring on P2, and a second adjustment to 4 pups (male pups) was made on P5. P0 was defined as the day of birth. On P21, the pups were separated from the dams. For this study, only male offspring rats were used, and they were assigned according to the experimental group that corresponded to them (NL or SL) and the type of diet provided from postnatal week (W) 3 to W11: standard diet (SD) (LabDiet 5001, cat #0001319: LabDiet), hypercaloric diet (HD) (made according to the formulation of the Envigo TD.88137 diet) or hypercaloric diet with 10% functional food (HDFF). In this way, the following groups were formed (6 to 8 rats in each): NLSD, NLHD, NLHD_FF_, SLSD, SLHD, and SLHD_FF_. To avoid any negative effects caused by social isolation, the rats were placed at a rate of 2 animals per cage, and measurements of body weight and weekly energy intake were taken.

### 2.3. Preparation of Experimental Diets

The hypercaloric diet (HD) and the hypercaloric diet with functional food (HDFF) were prepared from MP Biomedicals brand ingredients according to the formulation shown in Table 1. For the HDFF diet, the functional food (FF) was confected by mixing 90 g of amaranth flour (*Amaranthus hypochondriacus* L.), 5 g of ground chia seeds (*Salvia hispanica* L.), and 5 g of dissolved ethanolic extract (1 g/mL) of turmeric (*Curcuma longa* L.) of the Terana brand containing 1.5% curcumin. In the HD, 100 g of starch was substituted with the prepared FF; thus, the amount of FF in the HDFF was 100 g/kg diet (10%).

### 2.4. Turmeric Ethanolic Extract

An amount of 100 g of Terana brand turmeric powder was macerated with 700 mL of ethanol for 90 min with constant stirring. The mixture was filtered, and the supernatant was distilled under reduced pressure using a rotary evaporator (PRENDO, model 1750 SEVMEXICO, Puebla, Mexico) at a maximum temperature of 45 °C. The concentration of curcumin present in the extract was determined using gas chromatography coupled with mass spectrometry (GC-MS) at the Center for Nanosciences and Micro Nanotechnologies of the National Polytechnic Institute (CNMNIPN). In brief, ESI-Q-TOF-MS measurements were calculated using a micrOTOF-Q (Bruker Daltonics Inc., Billerica, MA, USA) mass spectrometer equipped with an automatic syringe pump from KD Scientific for sample injection. The ESI-Q-TOF mass spectrometer ran at 4.5 kV at a desolvation temperature of 180 °C. The mass spectrometer operated in the positive ion polarity. The ESI-Q-TOF-MS instrument was calibrated in the 50–3000 *m*/*z* range using an internal calibration standard (Tunemix solution). Data were processed via Bruker Data Analysis software version 4.1.

### 2.5. Intraperitoneal Glucose Tolerance Test

At the start of W11, an intraperitoneal glucose tolerance test was performed. Blood glucose concentration was determined after a 12 h fast with a glucometer (Accu-Chek Performa, Roche, Mannheim, Germany). A dose of 2.5 g/kg of body weight of glucose was administered intraperitoneally (Ip) from a 30% glucose solution, and glycemia was measured at 15, 30, 60, and 120 min after glucose administration. Regarding the glycemic values, the area under the glycemic curve (AUC) was calculated using the following formula:

$$AUC=\frac{{(G}_{t15}+G_{t0})(15-0)}{2}+\frac{{(G}_{t30}+G_{t15})(30-15)}{2}+\frac{{(G}_{t60}+G_{t30})(60-30)}{2}+\frac{{(G}_{t120}+G_{t60})(120-60)}{2}$$

where *G_t_* is the blood glucose of the rat at a given time.

### 2.6. Tissue Sampling

At the end of week 11, the rats were fasted for 12 h and anesthetized with sodium pentobarbital at a dose of 35 mg/kg Ip. A total of 5 mL of blood was collected using cardiac puncture, the animals were euthanized with an overdose of sodium pentobarbital, and dissection was subsequently performed in the abdominal region to remove the liver and retroperitoneal and epididymal white adipose tissue depots. The tissues were weighed, and their percentage relative to body weight was calculated. Blood samples were centrifuged at 2000× *g* at 4 °C for 15 min, and the serum was isolated and stored at −20 °C until further analysis of leptin, insulin, triglyceride, and cholesterol concentrations. After determining the percentage of liver weight, it was stored at −70 °C for subsequent analysis of triglyceride and cholesterol content.

### 2.7. Blood Biochemistry

Leptin and insulin concentrations were determined via the ELISA technique using specific enzymatic kits of the Merck brand: Millipore #EZRMI-13K for insulin determinations and #EZRL-83K for leptin determinations. Total triglyceride and cholesterol concentrations were quantified with the use of specific Randox brand reagent kits, following the supplier’s protocols in all cases.

### 2.8. Determination of Liver Lipids

An amount of 100 mg of liver tissue was weighed and homogenized in an Eppendorf tube with 0.5 mL of cold PBS buffer using a BioMasher II plunger. To the homogenates, 1 mL of chloroform/methanol 2:1 *v*/*v* was added, and they were stirred in a vortex for 1 min and left to stand for 12 h at a temperature of 5 °C. The tubes were centrifuged (4000 rpm, 4 °C, 15 min), the organic phase was separated, and the solvent was removed, leaving the samples to stand for 24 h in an extraction hood. The residue was dissolved with 1 mL of absolute ethanol, and total triglycerides and cholesterol were quantified using Randox kits.

### 2.9. Locomotor Activity Test in Open Field

At week 9, a locomotor activity test was performed. On the day of the test, the rats were habituated to the experimentation room for 30 min with white background noise and 220 lx illumination. Each rat was placed in an acrylic box with the dimensions 42 cm high, 62 cm long, and 49 cm wide, without a lid, and with a transparent base and black walls for 5 min. Time spent in ambulatory activity; time spent in non-ambulatory activity, which includes sniffing and grooming (stereotypical behavior); time spent resting; and the number of events of rearing events were measured.

### 2.10. Memory and Learning Test in the Barnes Maze

At week 10, a memory and learning test was performed in the Barnes maze, which is a flat, circular board (122 cm in diameter) with 18 equidistant holes located on the periphery. One of these holes contains an escape box (EB), where rats can enter and hide from the environment. On the walls of the experimentation room, there were visual signals that served as a reference for the rat to learn where the EB was located. Each rat was placed in the center of the board, and the time it took to find the EB (latency time) and the number of errors (sniffing in the other holes) were measured over a maximum of 5 min for 7 consecutive days (learning phase). On day 8 (retention phase), the EB was removed and a hole similar to the others replaced it, and the time spent by the rat in the quadrant where the EB was located was measured for 5 min.

### 2.11. Statistical Analysis

The mean of the data ± the standard error, or the median with the minimum and maximum points, are shown in the graphs. Data were analyzed with statistical software (SigmaPlot 12.0). Data normality was analyzed using the Shapiro–Wilk test. A two-way analysis of variance (ANOVA) or three-way ANOVA for repeated measures was used on the parametric variables with the Student–Newman–Keuls test for multiple comparisons, whereas the Kruskal–Wallis test was used on the non-parametric variables. A significant statistical difference was established at *p* < 0.05.

## 3. Results

### 3.1. Curcumin Content in the Turmeric Ethanolic Extract

**Figure 1:**
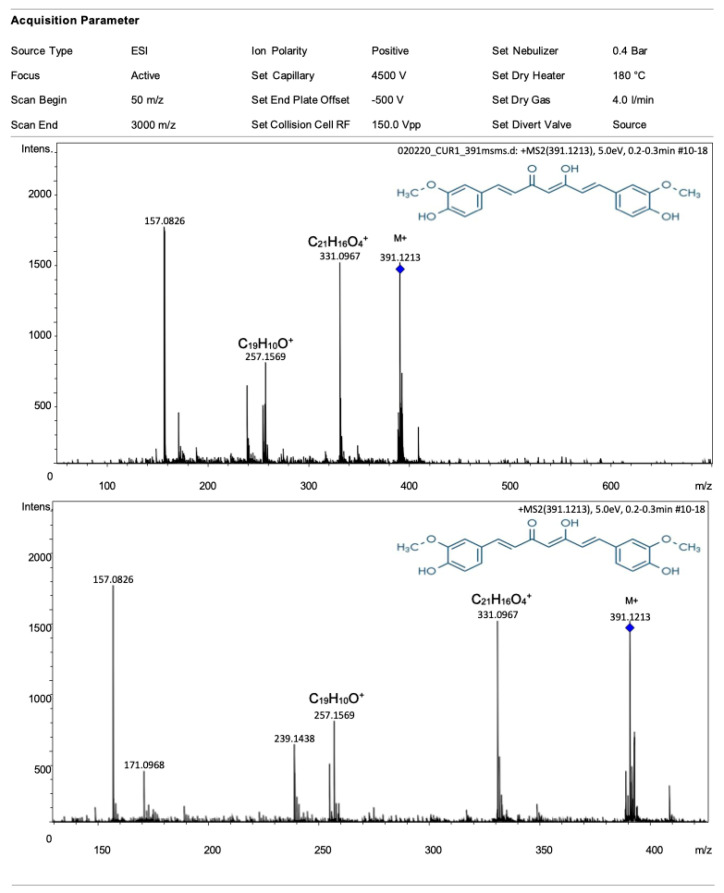
ESI-Q-TOF mass spectrum of turmeric ethanolic extract (**top**), zoom into a mass spectrum (**bottom**). The spectrum shows the expected M+ ion of curcumin (*m*/*z* 391) (blue rhomb). In addition, some ion peaks of other curcumin fragments are also observed.

The ESI-Q-TOF mass spectrum of the turmeric ethanolic extract is presented in Figure 1. The spectrum shows the expected M+ ion of curcumin (*m*/*z* 391) and other fragment ion peaks of curcumin, C_21_H_16_O_4_^+^ (*m*/*z* 331) and C_19_H_11_O^+^ (*m*/*z* 257). The quantitative analysis showed that the ethanolic extract of concentrated turmeric contained approximately 1.5% of curcumin.

### 3.2. Body Weight, Energy Intake and White Adipose Tissue

**Figure 2:**
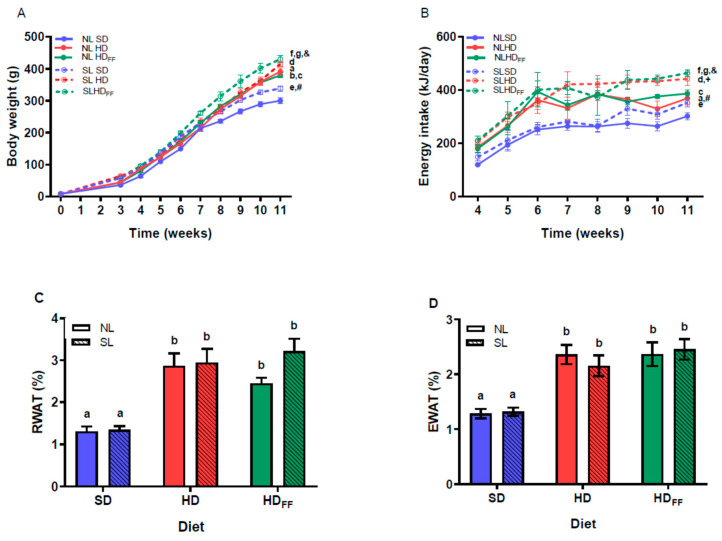
Homeostatic energy balance: (**A**) body weight, (**B**) energy intake, (**C**) retroperitoneal white adipose tissue weight (RWAT), and (**D**) epididymal white adipose tissue weight (EWAT). Data are expressed as mean ± standard error, *n* = 6–8 in each group. In (**A**,**B**), for a–g, *p* < 0.05 between groups within the same week (W): a, NLHD vs. NLSD W4 and W9–W11 on (**A**), W4 and W9–W11 on (**B**); b, NLHD_FF_ vs. NLSD W9–W11 on (**B**); c, NLHD_FF_ vs. NLHD W11 on (**A**,**B**); d, SLHD vs. SLSD W10 and W11 on (**A**), W4–W11 on (**B**); e, SLHD_FF_ vs. SLSD W10–W11 on (**A**,**B**); f, SLHD_FF_ vs. SLHD W11 on (**A**,**B**); g, SLHD_FF_ vs. SLHD W11 on (**A**,**B**). #, SLSD vs. NLSD W4 and W9–W11 on (**A**,**B**); +, SLHD vs. NLHD W9–W11 on (**B**); and &, SLHD_FF_ vs. NLHD_FF_ W4 and W9–W11 on (**A**) and W7 and W9–W11 on (**B**). Different literals mean a significant difference (*p* < 0.05) between diets on (**C**,**D**). NL = normal litter; SL = small litter; SD = standard diet; HD = hypercaloric diet; HD_FF_ = hypercaloric diet with functional food.

The reduction in the litter size (SL) produced an increase in body weight compared with the weight of the rats nursed in litters of normal size (NL) (Figure 2A), and this weight difference was significant on the weaning day (P21 or W3). Subsequently, from W3 to W11, the SL and NL rats were fed with one of the following study diets: the standard diet (SD), the hypercaloric diet (HD), or the HD with functional food (HDFF). The SL rats were heavier than the NL rats for each of the study diets. In the NL and SL rats, feeding with the HD and the HDFF produced a significant increase in body weight in the NL rats in W4 and W8–W11 and in the SL rats in W10–W11. The SL rats fed with the HD that also contained functional food (HDFF) were heavier than the SL rats fed with the HD. This increase in body weight was not observed in the NL rats fed with the HDFF when compared with those rats fed with the HD (Figure 2A). These changes in body weight were related to increases in energy intake. Similarly, the SL rats showed a higher energy intake than the NL rats for the three diets in W9–W11. The rats fed with the HD showed a higher energy intake than the rats fed with the SD (W4–W11), and the energy ingestion of the rats fed with the HDFF was higher than that of the rats fed with the HD in W10–W11 (Figure 2B).

Additionally, the percentage weight of the retroperitoneal and epididymal white adipose tissue depots (RWAT and EWAT, respectively) in W11 were higher in all the groups fed with the HD or HDFF when compared with the rats fed with the SD (Figure 2C,D). The addition of the functional food to the HD did not modify the percentage weight of RWAT or EWAT shown by the SLHD_FF_ rats, but the NLHD_FF_ rats showed a non-significant decrease in the mass of this fat deposit.

### 3.3. Relative Liver Weight and Lipid Content in Serum and Liver

**Figure 3:**
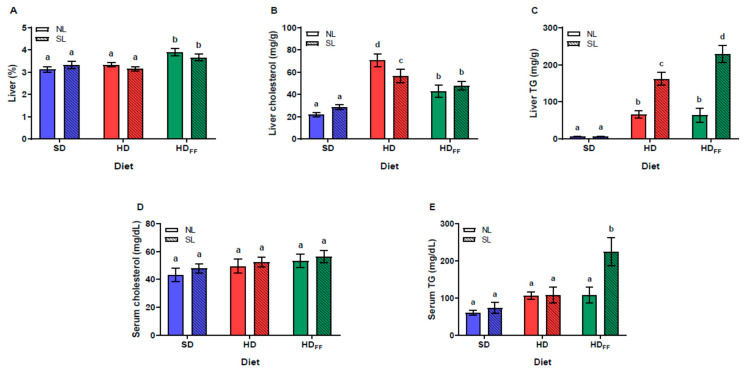
Lipid metabolism: (**A**) relative liver weight (%), (**B**) liver cholesterol, (**C**) liver triglycerides (TG), (**D**) serum cholesterol and (**E**) serum triglycerides (TG). Data are expressed as mean ± standard error, *n* = 6–8 in each group. Different literals mean a significant difference (*p* < 0.05) between groups.

The reduction in litter size did not affect the relative liver weight. Only the rats fed with the HDFF from both litter sizes showed a higher relative weight in comparison with the SD and HD rats. Furthermore, the liver percentage weight of the HD rats was not different from that of the SD rats (Figure 3A). Liver cholesterol (Figure 3B) was also not affected by the reduction in litter size, but the cholesterol content was significantly increased in the groups of rats fed with the HD; interestingly, FF decreased liver cholesterol in the NL and SL rats. The liver triglycerides (Figure 3C) significantly increased in the HD and HDFF rats. This increase was notably higher in the SL rats than in the NL rats. Serum cholesterol (Figure 3D) and serum triglycerides (Figure 3E) were not affected by the reduction in litter size or feeding with the high-fat diet. Feeding the HDFF to the SL rats produced a significant increase in serum triglycerides in comparison with all other groups.

### 3.4. Glucose Homeostasis

**Figure 4:**
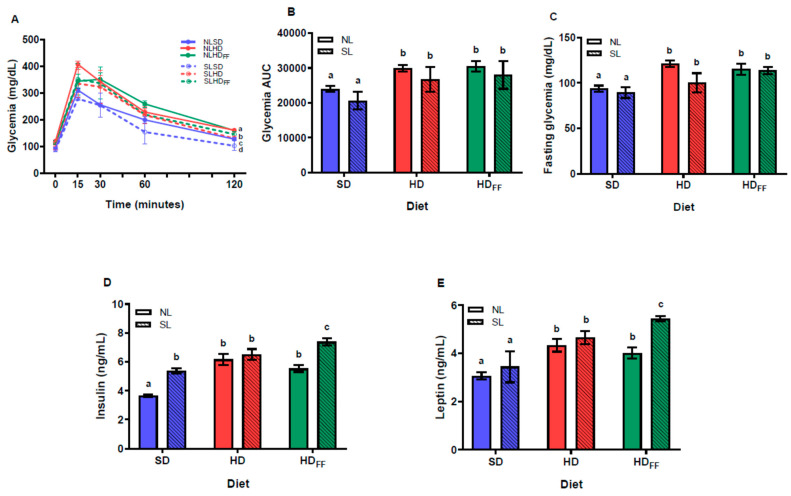
Glucose homeostasis: (**A**) glycemia during the IP glucose tolerance test, (**B**) area under the curve (AUC) of glycemia, (**C**) fasting glycemia, (**D**) insulin serum levels, and (**E**) leptin serum levels. Data are expressed as mean ± standard error, *n* = 6–8 in each group. In (**A**), for a, b, c, and d, *p* < 0.05 between groups within the same time (t): (a), SLSD vs. NLSD t60–t120; (b), SLHD vs. NLHD t0 and t120; (c), NLHD vs. NLSD t0, t30 and t120; d, SLHD vs. SLSD t120. Different literals mean a significant difference (*p* < 0.05) between groups (**B**–**E**).

Although the reduction in litter size did not affect the fasting glycemia (Figure 4C) nor glycemia during the intraperitoneal glucose tolerance test (IP-GTT) (Figure 4A), insulin serum levels significantly increased in the SL rats (Figure 4D). However, HD and HDFF rats showed an increased glycemia on several points of the curve during the IP-GTT, including fasting glycemia, which was better observed on the area under the curve (AUC) of the glycemia graph (Figure 4B). This shows that all the groups of rats fed with the HD or the HDFF (NLHD, NLHD_FF_, SLHD, and SLHD_FF_) showed higher values of glucose AUC (Figure 4B). Insulin serum levels were higher in the SLSD group compared with the NLSD group and even higher in the HD rats. Moreover, the increment in serum insulin shown by the SL rats fed with the HDFF were the highest levels of serum insulin (Figure 4D). Serum leptin levels were also increased in the HD rats. Feeding the HDFF to the SL rats (SLHD_FF_) produced an increase in leptin serum levels with respect to NLHD_FF_. Interestingly, the insulin and leptin levels were significantly lower in the NL rats fed with the HDFF compared with those of the SLHD_FF_ rats (Figure 4E).

### 3.5. Locomotor Activity in the Open Field Test

**Figure 5:**
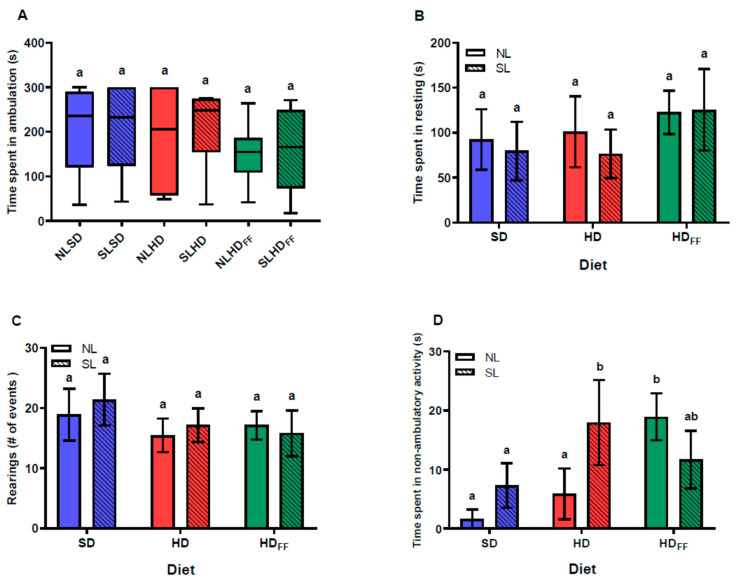
Locomotive behavior in open field: (**A**) time spent in ambulation, (**B**) time spent resting, (**C**) number of rearing events, and (**D**) time spent in non-ambulatory activity. Data are expressed as median and minimum to maximum points (**A**) and mean ± standard error (**B**–**D**); *n* = 6–8 in each group. Different literals mean a significant difference (*p* < 0.05) between groups.

There were no differences among the groups with regard to locomotion and resting times or in the number of rearing behavior events (Figure 5A–C). There was no difference resulting from the litter size in the SD regarding non-ambulatory activity (i.e., grooming) time, but the rats from the SL fed with the HD spent more time on grooming compared with the NLHD and SD rats. However, the grooming time of the HDFF rats was longer compared with that of the SD rats (Figure 5D).

### 3.6. Memory and Learning Test on Barnes Maze

**Figure 6:**
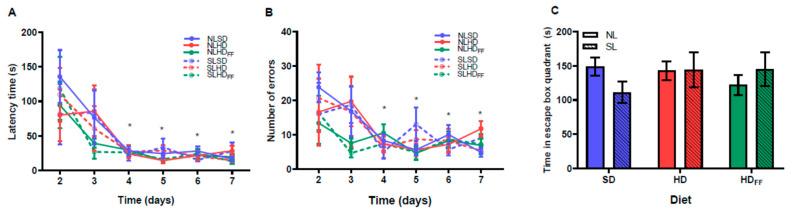
Memory and learning test on Barnes maze: (**A**) latency time, (**B**) number of errors, and (**C**) time spent in the escape box quadrant. Data are expressed as mean ± standard error; *n* = 6–8 in each group. * *p* < 0.05, the main significant differences with respect to days 2 and 3 on (**A**,**B**). (**C**) No significant differences were found.

The cognitive performance did not differ between the groups (Figure 6A–C). All the rats learned where the escape box was placed from day 4, and from this day on, they reduced their latency time and number of errors in a similar way (Figure 6A,B). Additionally, there were no differences between the groups in terms of the time expended in the quadrant where the escape box was placed (Figure 6C).

## 4. Discussion

Obesity is a worldwide health problem, with a prevalence that has tripled since 1975, and is a risk factor in multiple non-communicable chronic diseases such as type 2 diabetes mellitus, cardiovascular and cerebrovascular diseases, mental disorders, hypertension, dyslipidemias, non-alcoholic hepatic steatosis, and some types of cancer [2]. The research objective was to study the possible beneficial effects of supplying a functional food against the alterations in metabolism and behavior produced by obesity. This functional food made with amaranth flour, chia seed, and curcumin extract was fed, as part of a hypercaloric diet, to a rat offspring model of childhood/juvenile obesity since both neonatal overfeeding and a high-fat diet produce medium-term changes such as early diabetes and accelerated aging.

The results of the present work confirm that a reduction in litter size produces a significantly higher body weight gain in rats [11,31] or mice at weaning [9]. Furthermore, feeding the SL rats with the HD after weaning also significantly increased the body weight and energy intake of the animals at the end of the experiment, supporting the use of this model to study childhood obesity [10]. The percentage weight of both visceral adipose tissue depots, RWAT and EWAT, considered markers of adiposity, were higher in the animals fed with the HD or HDFF but were not affected by the reduction in litter size.

The higher body weight and food intake shown by the offspring rats supports the notion that the higher milk availability that occurs as a result of reducing the litter size alters the normal development of the brain circuits that control food intake [32] and the secretion of hormones that determine the metabolic routes. An animal with early overnutrition shows adaptive responses in the endocrine system and metabolism by programming the development of obesity and metabolic disorders in adulthood [12]. These changes are exacerbated when the offspring are fed a high-calorie diet, resulting in persistent overweight and early obesity in juvenile age, as well as other disorders such as hyperleptinemia, hyperinsulinemia, glucose intolerance, and hepatic steatosis, among other effects. Unexpectedly, the group of rats with childhood obesity that was also fed the functional food in the high-fat diet (SLHD_FF_) showed further body weight gain and energy intake when compared with the HD rats from both litter sizes. The HDFF contained one percent more lipids than the HD. Although this was only a slight difference, it may explain, at least in part, the higher body weight gain shown by the SLHD_FF_ rats compared with the SLHD rats and suggests that the groups of rats coming from small litters and fed with the HD or HDFF showed long-lasting alterations in the control of food intake.

In the liver, cholesterol and TG concentrations were increased in the rats fed with HD. Similar findings were reported by [10], where offspring reared in small litters and fed for 16 weeks with a high-fat diet (lard) showed higher liver weight, hepatic triglycerides, and steatosis score, suggesting that an increase in liver lipogenesis could be involved. Conversely, the rats fed with the HDFF from both litter sizes showed a lower hepatic cholesterol content, whereas liver TG significantly increased in the SL rats and did not change in the rats from the NL when compared with the values of the rats fed with HD, suggesting that FF was able to decrease cholesterol and did not modify the TG content in the liver of the rats raised in the NL.

Ji et al. [10] also reported that, at week 10, the SL rats showed similar triglyceride and cholesterol serum levels and were beginning to show higher adipose tissue depots (RWAT and EWAT) than the NL rats. In our experiment, at postnatal week 11, the SL animals, in comparison with the NL animals, showed similar RWAT and EWAT weights. Similar to our results, in theirs, the SL rats fed with the hypercaloric diet showed higher RWAT and EWAT weights and liver TG levels. The addition of the FF to the HD was expected to decrease the obesity markers, but, unexpectedly, in our study, the increases in energy intake, body weight gain, hepatic weight, liver cholesterol, and serum TG levels were exacerbated only in the group of SL rats fed with the HDFF.

Glycemia homeostasis was negatively affected by the HD and the HDFF in both the NL and SL rats, but the insulin and leptin levels were differently affected by the HDFF. The highest insulin and leptin serum levels were found in the rats from the SL fed with the HDFF. In contrast, the rats raised in the normal litters (10 pups per mother) and fed with the HDFF diet had lower levels of insulin and leptin compared with those of the HD rats of both litter sizes.

In the final week, the rats from the NLHD_FF_ group showed a significant decrease in body weight gain and liver cholesterol and non-significant decreases in RWAT and leptin and insulin serum levels compared with the NLHD rats. Only the time spent in non-locomotive activity was significantly higher in the NLHD_FF_ rats. Overall, these results suggest that, in the NL rats, the FF produced some of the expected improvements in the obesity markers.

The ingestion of a high-fat diet during early life (P21–P60) in mice produces neuroinflammation, with increased expression of TNFα and IL-1β, and alterations in hippocampus neurogenesis and spatial memory, as evaluated by a lower discrimination index in the novel object location recognition (NOR) test without showing locomotive alterations [33]. Similarly, it has been reported that early-life overfeeding owing to a reduced litter size in rats [34] or NMRI mice alters spatial memory and increases anxiety-like behaviors without alterations in ambulatory activity or the number of rearing events in later life [35]. In the present study, a reduction in litter size or feeding the offspring with high-fat diets did not significantly alter the learning or spatial memory evaluated in the Barnes maze. However, the locomotive behavioral test revealed that the time spent in non-ambulatory activity significantly increased in the groups that were fed with a high-fat diet, with or without functional food. Although non-significantly altered, the ambulation time of the groups fed with the HDFF showed a tendency to be lower, and the opposite was observed for the immobility time, revealing the tendency of these groups to spend more time resting when compared with the SD groups.

Non-ambulatory activity in rats includes postural adjustments and stereotyped behaviors such as sniffing and grooming. Stereotyped movements have been related to low-grade chronic inflammation that manifests in the central nervous system, especially in the comorbidities of fatty liver, obesity, and diabetes mellitus, owing to excess production of proinflammatory cytokines [36]. Stereotyping is also present when there are abnormalities in the circuits of the basal ganglia [37], including the dopaminergic motivation system, producing stereotyped movements in individuals with little impulse control. These suggest that childhood overnutrition combined with a diet high in sugars and fats produces neuroinflammation, impairing functioning and reinforcing addictive behaviors in the face of pleasant stimuli, such as the intake of sugary hypercaloric diets, manifesting in stereotyped movements. Here, the HD increased non-ambulatory activity time, but only in the SLHD rats; furthermore, the NLHD_FF_ group also presented a marked increase in non-ambulatory activity time in comparison with the NLSD and NLHD groups. Therefore, the combination of amaranth, chia, and curcumin with a hypercaloric diet was found to be detrimental to the functioning of the central nervous system since feeding the HD to the NL rats did not produce changes in the behavioral tests. The hypercaloric diet itself in that period of time was unable to generate such alterations at the central level of the rats from the NL.

In the memory and learning tests, there were no alterations in any study group, possibly because the Barnes maze test is less sensitive than the NOR or Morris maze tests in evaluating the alterations in memory and learning produced in this animal model. Another possibility is that the experiment was conducted when the damage began to manifest at the central level since the offspring fed with a hypercaloric diet spent a longer time performing non-ambulatory movements in the locomotor activity tests. Thus, we suggest that the comorbidities caused by being overweight occur first, the metabolic disturbances were followed by behavioral and, finally, cognitive alterations. Previous studies have supported the notion that resistance to leptin and insulin at the central level damages the circuits associated with memory and learning by reducing trophic factors in the brain, with a decrease in neurogenesis, synaptogenesis, and synaptic plasticity [38]. The hippocampus also has receptors for leptin, and leptin resistance could alter the synaptic plasticity of this structure, making cognitive deficits prevalent; however, for the latter to occur, chronic stages could be needed in individuals with obesity.

It has been reported that some substances and functional foods produce beneficial effects on health that oppose the alterations produced by obesity. In this study, supplementation with amaranth seed in the diet was proposed as a possible treatment for obesity comorbidities since pseudo-cereal grains such as buckwheat, amaranth, and quinoa are rich in a wide range of compounds such as flavonoids, phenolic acids, trace elements, fatty acids, and vitamins. These compounds are known to have an effect on human health such as the prevention of, and reduction in, many degenerative diseases [39], among the other benefits they provide. Other substances have also been found that not only help to reduce body weight but also focus on treating non-alcoholic hepatic steatosis, for example, omega-3 fatty acids, which improve the lipid profile, glucose, and circulating triglycerides. Among these are foods that contain alpha-linoleic acid (ALA) that can diminish body weight gain [40]. Chia seed fat is composed of more than 60% ALA [18], which can be converted into n-3 LCPUFAs, the latter being capable of preventing various metabolic biochemical disorders [41]. In a previous study, the ingestion of a diet enriched with sucrose produced a higher body weight gain, which was not reduced by the addition of chia seed flour (36%) to the same diet for three months; nevertheless, the animals showed a lower body mass index and visceral adiposity index [24]. It has been reported [25] that the ingestion of a diet supplemented with 1% or 2% curcumin for 10 weeks after weaning decreased the body weight gain of offspring rats reared in small litters by increasing the expression of UCP-1, energy expenditure and mRNA levels of beta 3 adrenergic receptors in subcutaneous fat and restored normal glucose tolerance and circulating lipids levels. In our experiment, the HDFF that contained buttermilk (20%), amaranth, chia and curcuminoid extract produced in the SL rats unexpected further increases in energy intake, body weight gain, hepatic and circulating triglycerides, leptin, and insulin serum levels, and time engaged in non-ambulatory movements compared with the rats fed with the HD, suggesting that the animals liked this diet more, thus producing a further energy intake and causing the exacerbation of the alterations produced by the overfed state.

Recent reviews of the effects of curcumin on body weight in human trials that were performed at widely variable doses (between 70 and 2400 mg/day) concluded that curcumin intake could reduce body mass index, body weight, and waist circumference [26]; however, other studies conducted in human or animal models of obesity found that there is not sufficient information to support a body weight-reducing effect [27]. One possible explanation is that the bioavailability of curcumin is low because of its poor gastrointestinal absorption and rapid metabolism [42]. In this study, the used dose was low compared with those reported in previous studies, and its combination with chia and amaranth made a palatable and anabolic food.

There are few studies on rat models of childhood obesity that combine a reduction in litter size and, after weaning, feeding with an HD, which is a common perturbance present in the human child. In this model, it has been demonstrated that early overnutrition induced by a reduction in litter size produces a metabolic programming of obesity, affecting the hormones and hypothalamic circuits that control food intake. When, posteriorly, these rats are fed with an HD, the disturbances are exacerbated [9]. To our knowledge, this is the first study conducted on rats from this model, providing a functional food in the diet with the aim of reducing obesity.

The use of functional foods may not be adequately validated by scientific data on their safety, efficacy, and effect on health and/or pathological conditions. This is in part owing to the lack of in vivo research confirming the supposed beneficial health effects in specific pathological conditions [43]. Therefore, research such as that carried out in the present study on the risks and benefits of functional foods in individuals with negative metabolic programming and a high-calorie diet is of special importance. Further studies are needed to investigate if functional foods made with the extracts of amaranth, chia seeds, and turmeric produce greater beneficial effects in the model of childhood obesity.

## 5. Conclusions

In the childhood/juvenile obesity rat model used here, the SL, in combination with the HD, produced metabolic alterations and anxiety-like behaviors; however, the provision of a functional food made with amaranth flour, chia seed, and curcumin failed to reduce or prevent these alterations, and an exacerbation was even observed in some metabolic indicators. However, in the NL rats, the functional food produced some of the expected improvements in health, such as significant decreases in body weight gain and liver cholesterol and non-significant decreases in RWAT, leptin, and insulin serum levels.

## Figures and Tables

**Table 1:** Chemical and energy composition of the different study diets.

	SD	HD	HDFF
Carbohydrates	48.7%	49.3%	46.7%
Lipids	13.4%	21.0%	22.0%
Protein	24.1%	19.5%	21.1%
Fiber	5.2%	5.0%	5.0%
Minerals	6.9%	3.5%	3.5%
Vitamins	1.7%	1.7%	1.7%
Energy content	12.6 kJ/g	19.43 kJ/g	19.34 kJ/g

SD = standard diet; HD = hypercaloric diet; HDFF = hypercaloric diet with functional food (FF). The FF was composed of 9% amaranth, 0.5% chia, and 0.5% ethanolic extract of turmeric.

## Data Availability

The original contributions presented in the study are included in the article, further inquiries can be directed to the corresponding author.
